# Diagnostic value of endobronchial ultrasound elastography combined with rapid onsite cytological evaluation in endobronchial ultrasound-guided transbronchial needle aspiration

**DOI:** 10.1186/s12890-021-01748-4

**Published:** 2021-12-20

**Authors:** Jing Huang, Yuan Lu, Xihua Wang, Xiaoli Zhu, Ping Li, Jing Chen, Pingsheng Chen, Ming Ding

**Affiliations:** 1 Department of Respiratory and Critical Care Medicine, School of Medicine, Zhongda Hospital, Southeast University, Dingjiaqiao 87#, Nanjing, Jiangsu China; 2 Department of pathology and pathophysiology, School of Medicine, Southeast University, Dingjiaqiao 87#, Nanjing, Jiangsu China

**Keywords:** Endobronchial ultrasound (EBUS) elastography, Rapid onsite cytological evaluation (ROSE), Endobronchial ultrasound-guided transbronchial needle aspiration (EBUS-TBNA)

## Abstract

**Background:**

Endobronchial ultrasound (EBUS) elastography has been used in EBUS-guided transbronchial needle aspiration (EBUS-TBNA) to identify malignant lymph nodes based on tissue stiffness. Rapid onsite cytological evaluation (ROSE) has been widely utilized for onsite evaluation of sample adequacy and for guiding sampling during EBUS-TBNA. The aim of this study was to investigate the diagnostic value of combined EBUS elastography and ROSE in evaluating mediastinal and hilar lymph node status.

**Methods:**

Retrospective chart review was performed from December 2018 to September 2020. Patient demographics, EBUS elastography scores, and ROSE, pathologic, and clinical outcome data were collected. The EBUS elastography scores were classified as follows: Type 1, predominantly nonblue; Type 2, partially blue and partially nonblue; and Type 3, predominantly blue. A receiver operating characteristic curve was used to compare the sensitivity, specificity, positive predictive value, negative predictive value, positive likelihood ratio, and negative likelihood ratio for evaluation of malignant lymph nodes among the EBUS elastography, ROSE, and EBUS combined with ROSE groups.

**Results:**

A total of 245 patients (345 lymph nodes) were included. The sensitivity and specificity of the EBUS elastography group for the diagnosis of malignant lymph nodes were 90.51% and 57.26%, respectively. The sensitivity and specificity in the ROSE group were 96.32% and 79.05%, respectively. The sensitivity, specificity, positive likelihood ratio, and negative likelihood ratio of EBUS elastography combined with ROSE were 86.61%, 92.65%, 11.78, and 0.14, respectively, and the area under the curve was 0.942.

**Conclusions:**

Combining EBUS elastography and ROSE significantly increased the diagnostic value of EBUS-TBNA in evaluating mediastinal and hilar lymph node status compared to each method alone.

**Supplementary Information:**

The online version contains supplementary material available at 10.1186/s12890-021-01748-4.

## Background

Endobronchial ultrasound-guided transbronchial needle aspiration (EBUS-TBNA) technique was developed in 2002. Currently, EBUS-TBNA is highly recommended by the National Comprehensive Cancer Network (NCCN) and the American College of Chest Physicians (ACCP) for diagnosis and preoperative staging of patients with lung cancer. Ultrasonography is frequently used to identify malignant lymph node status and guiding lymph node aspiration. However, conventional ultrasonography has not shown particularly high accuracy in identifying malignant lymph nodes [1]. Ultrasound elastography is a real-time imaging technique to identify malignancy based on the tissue stiffness. The relative elasticity or stiffness of the tissue is determined by applying stress to the tissue, causing deformation; the resulting echo signals are converted to real-time images [2]. Red signals indicate a low elasticity coefficient in tissues, while blue signals demonstrate a high elasticity coefficient in tissues. Compared to benign lesions, malignant lesions are usually firmer, making them easier to detect with elastosonography [3]. Recently, ultrasound elastography has been widely applied for diagnosis of breast cancer and prostate cancer and in liver fibrosis staging [4–6]. Since EBUS elastography can rapidly identify malignant mediastinal lymph nodes, it has also been used in EBUS-TBNA to better identify suspicious lesions. Mittal et al. reported that the sensitivity and specificity of EBUS elastography to identify malignant lymph nodes were 85.7% to 100% and 66.7% to 92.3%, respectively [7].

During EBUS-TBNA, rapid onsite cytological evaluation (ROSE) has been widely used to evaluate samples for example, guiding following passes and determining adequacy of samples for further immunohistochemistry or next generation sequencing [8]. However, some studies have shown that ROSE alone does not improve the diagnostic sensitivity of EBUS-TBNA [9].

Recently, ROSE has been used as an assistive diagnostic technique in some EBUS elastography related studies; however, the diagnostic value of combined EBUS elastography and ROSE remains undetermined [10, 11]. The aim of this study was to investigate the diagnostic value of combined EBUS elastography and ROSE in EBUS-TBNA.

## Materials and methods

### Patients

A retrospective study was performed to identify patients who underwent the EBUS procedure due to enlargement of mediastinal or hilar lymph nodes in a single institution from December 2018 to September 2020. This study was approved by the Ethics Committee of Zhongda Hospital, School of Medicine, Southeast University. Patients with mediastinal lymphadenopathy were included. Patient demographics, pathologic features, and clinical outcome data were collected by chart review. Lymph node samples were divided into three groups: EBUS elastography, ROSE, and combined EBUS elastography and ROSE.

Patient results from routine blood tests, chest high-resolution computerized tomography (CT) scans, and electrocardiograms were reviewed before the procedure. The patients were required to fast for 6–8 h before the procedure, and patients’ status was monitored by an anesthesiologist during the procedure. EBUS-TBNA was performed under conscious sedation combined with local anesthesia and the convex probe EBUS (BF-UC260F, Olympus, Japan) was inserted via the nasal route. After identifying the lesion, the size of lymph nodes and distribution of the vessels were evaluated,and the EBUS elastography images were analyzed by two pulmonologists individually. At least three passes were performed for each lesion with 15–20 aspirations. The 21 G needles (NA-201SX-4022, Olympus® Corporation, Japan) were used for aspiration.

### EBUS elastography image analysis

**Fig. 1:**
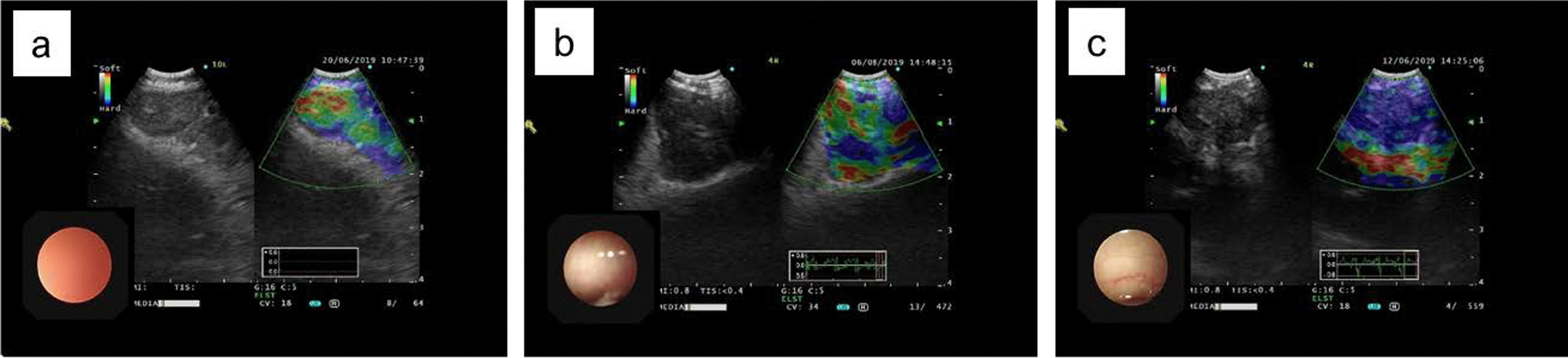
Representative lymph node status by EBUS elastography image analysis. **a** Type 1, predominantly nonblue (green, yellow, or red); **b** Type 2, partially blue, partially nonblue (green, yellow, or red); **c** Type 3, predominantly blue

Real-time EBUS B-mode was performed to evaluate the lesion and part of the normal tissue followed by elastography image analysis. During the procedure, the operator gently applied pressure to the convex probe to perform elastography, and the images were recorded for further analysis. The elastography images were divided into three categories according to the scoring system of Izumo et al. [12]: Type 1 (predominantly nonblue); Type 2 (partially blue and partially nonblue); and Type 3 (predominantly blue) (Fig. 1). After elastography image analysis, EBUS-TBNA aspiration was performed as usual. If a Type 2 elastography image was found in the lesion, the blue area was considered the preferred area for aspiration [13].

### ROSE procedure and interpretation

ROSE interpretation was performed by a single pulmonologist with at least one year of experience in evaluating ROSE results and who was blinded to the results of EBUS elastography image analysis. The specimens were collected, and smeared on a clean glass slides. The remaining specimens were fixed in 10% formalin and sent to the Pathology Department for routine testing. Diff-Quick staining was used for rapid staining in ROSE according to the manufacturer’s instructions [14]. Briefly, the slides were stained with solution A for 15–20 s, followed by solution B for an additional 15–20 s. The slides were then rinsed in tap water and dried with absorbent paper.

ROSE interpretation was performed according to the guidelines of the Papanicolaou Society of Cytopathology System for Reporting Respiratory Cytology. In general, the results were classified into five categories [15]: 1. non-diagnostic (unsatisfactory); 2. negative for malignancy; 3. atypical cells present; 4. suspicious for malignancy. 5. positive for malignancy. For subsequent studies, Categories 2 and 3 was defined as negative cases, while categories 4 and 5 was defined as positive cases. Category 1 was not included in the subsequent studies. The results of ROSE and elastography will be compared to the final pathology diagnosis from biopsy/resection specimen. For patients with negative results, additional ancillary tests will be performed to rule out other possibilities if clinically indicated. These patients will be followed up for at least three months.

### Statistics

SPSS (SPSS Inc., Chicago, IL, USA) statistical software, version 22.0 was used for statistical analysis. All quantitative data obtained are expressed as the mean ± standard deviation. Receiver operating characteristic (ROC) analysis was performed to determine the test performance of the different methods. The sensitivity, specificity, positive predictive value (PPV), negative predictive value (NPV), positive likelihood ratio, and negative likelihood ratio of EBUS elastography, ROSE, and EBUS elastography combined with ROSE for detecting malignant lymph nodes were compared. All *p* values were based on two-sided testing, where *p* values less than 0.05 were considered significant.

## Results

### Patient characteristics

**Fig. 2:**
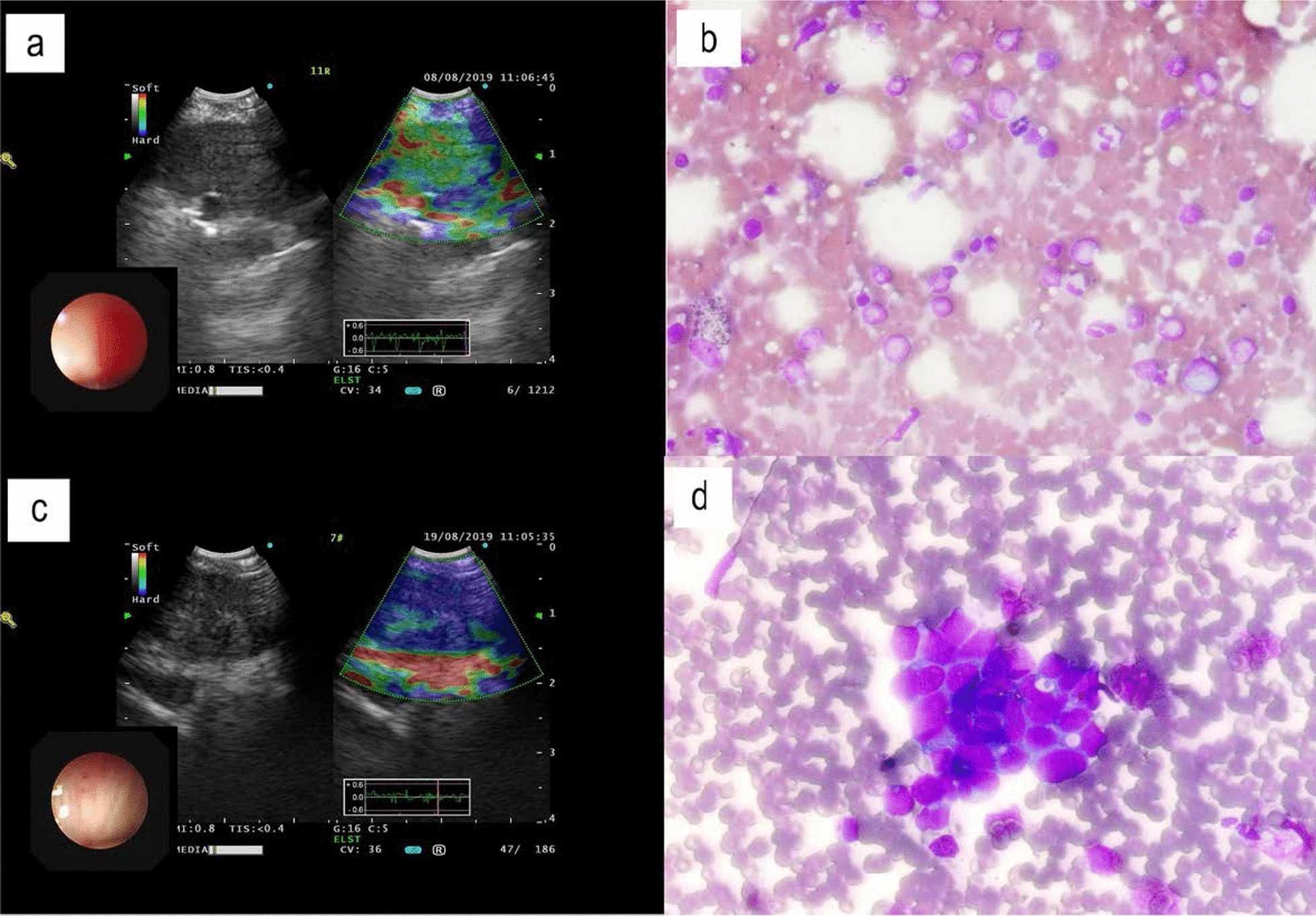
Representative lymph node status of Types 1 and 3 EBUS elastography images from 2 patients. EBUS elastography image (**a**) and ROSE (**b** Diff-Quik Stain, × 400) of 11R lymph node from a patient with inflammation; EBUS elastography image (**c**) and ROSE (**d** Diff-Quik Stain, × 400) of 7# lymph node from a patient with small cell carcinoma

From December 2018 to September 2020, a total of 247 patients were included. Two patients were excluded from the final analysis since there was no final pathological diagnosis. Patient characteristics and clinical information are summarized in Table 1. The mean age was 61.60 ± 11.85 years old (ranging from 26 to 86 years old), with a predominance of males (149 males, 96 females). A total of 147 cases were identified as positive for malignancy, while 99 cases were negative (Table 2). A total of 345 aspirated lymph nodes were included in the cohort, including 257 specimens with EBUS elastography results and 269 specimens with ROSE results. Among the 147 malignant cases, adenocarcinoma was the most common malignancy (n = 73, 49.66%), followed by small cell carcinoma (n = 26, 17.69%) and squamous cell carcinoma (n = 23, 15.66%). Among benign cases, sarcoidosis (n = 36, 36.36%), no abnormality identified (n = 35, 35.35%), and inflammation (n = 16, 16.16%) were the most common pathological diagnoses with EBUS-TBNA. One patient had small cell carcinoma and comorbid tuberculosis. Representative cases of benign and malignant lesions are shown in Fig. 2.

**Table 1:** Patient characteristics

	n = 245
Sex	
Male (%)	149 (60.81%)
Female (%)	96 (39.18%)
Age (years, Mean ± SD, range)	61.59 ± 11.85 (26–86)
Lymph node size(cm) (Mean ± SD, range)	2.27 ± 0.69 (0.8–4.7)
TBNA passes (Mean ± SD, range)	4.54 ± 1.02 (3–6)

cm, centimeter; SD, standard deviation; TBNA, transbronchial needle aspiration

**Table 2:** Final pathological results

TBNA Pathology (n = 245)	Number (%)
Malignant	147
Adenocarcinoma	73 (49.66)
Small cell carcinoma*	26 (17.69)
Squamous cell carcinoma	23 (15.66)
Undifferentiated/poorly differentiated carcinoma	18 (12.24)
Metastasis of breast cancer	2 (1.38)
Sarcoma	1 (0.69)
Diffuse large B-cell lymphoma	1 (0.69)
Multiple myeloma	1 (0.69)
Thymoma, type B3	1 (0.69)
Adenoid cystic carcinoma	1 (0.69)
Benign	99
Sarcoidosis	36 (36.36)
No abnormality identified	35 (35.35)
Inflammation	16 (16.16)
Tuberculosis*	8 (8.08)
Pneumoconiosis	3 (3.03)
Actinomycetes	1 (1.01)

TBNA, transbronchial needle aspiration

*One patient was diagnosed with small cell carcinoma comorbid with tuberculosis

### Comparison of results among EBUS elastography, ROSE, and combined EBUS elastography with ROSE groups

**Fig. 3:**
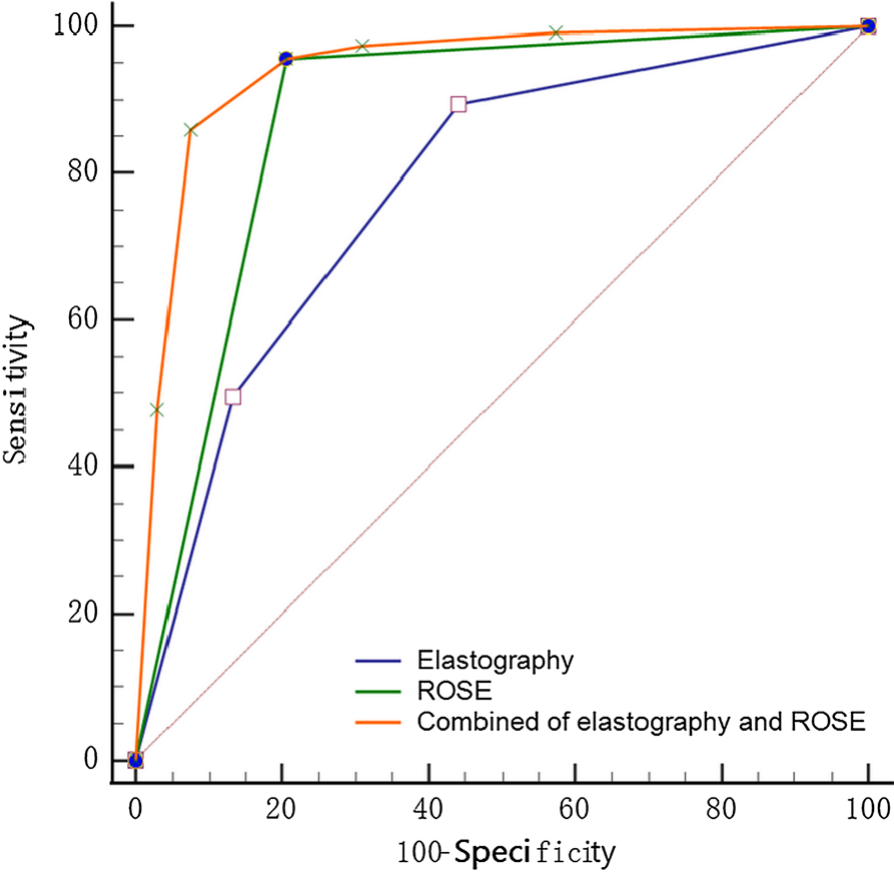
Comparison of ROC curves of EBUS elastography, ROSE and combined EBUS elastography and ROSE groups

As demonstrated in Table 3, the sensitivity and specificity of EBUS elastography alone in the diagnosis of malignant lymph nodes were 90.51% and 57.26%, respectively. The corresponding sensitivity and specificity in the ROSE group were 96.32% and 79.05%, respectively. The sensitivity and specificity in the combined group were 86.61% and 92.65%, respectively, with an area under the curve (AUC) of 0.942 which was greater than those of the only elastography (*p* < 0.001) or only ROSE groups (*p* = 0.0202). The combination of EBUS elastography and ROSE thus had the best diagnostic value among the three groups (Table 3, Fig. 3). The number of FN, FP, TP, TN results in three groups can be obtained from Additional file 1.

**Table 3:** Diagnostic performance of EBUS elastography, ROSE and combination

Group	Sensitivity (%)	Specificity (%)	PPV (%)	NPV (%)	+LR	−LR	AUC	Youden index
EBUS elastography	90.65	57.63	71.60	84.00	2.14	0.16	0.776	0.4827
ROSE	95.73	79.05	87.70	92.20	4.57	0.05	0.875	0.7478
Combination Group	85.84	92.65	95.10	79.70	11.67	0.15	0.940	0.7849

## Discussion

EBUS-TBNA has become a mature interventional diagnostic procedure in clinical practice. Previous studies have found no statistically significant difference in diagnostic sensitivity between EBUS-TBNA and mediastinoscopy for malignant lymph nodes evaluations, in cases where previous imaging results have indicated enlargement of mediastinal and/or hilar lymph nodes or adjacent lesions in the lungs [16]. However, compared to mediastinoscopy, EBUS-TBNA is less invasive, less expensive, and more tolerable for patients [17, 18]. EBUS-TBNA has gradually replaced mediastinoscopy and is currently recommended in multiple guidelines as the first choice for mediastinal staging in lung cancer.

To further improve diagnostic sensitivity of EBUS-TBNA and decrease procedure-related complications, some studies have analyzed echographyc features such as echogenicity (homogeneous or heterogeneous), margin status, presence or absence of central hilar structure, short axis diameter, and coagulative necrosis under B-mode ultrasound to evaluate lesions [1, 19, 20]. Even when including such features with EBUS, discrepancies have persisted among different studies [21]. To date, no EBUS features have proven to be consistent with the diagnosis of malignant lymph nodes. Thus, combining real-time imaging technology and EBUS elastography could be a new technique to resolve these problems. Ultrasound elastography is increasingly widely applied in the diagnosis of breast cancer, prostate cancer, liver disease, myopathies, etc. [22–25].

Endoscopic elastography has been well developed recently and has demonstrated its superiority in lesion evaluation compared with conventional ultrasound, especially in esophageal endoscopic ultrasound. A previous study showed that the sensitivity, specificity, PPV, NPV, and overall accuracy of endoscopic elastography for diagnosing pancreatic solid tumors were 93%, 66%, 92%, 69%, and 85%, respectively [26], while the sensitivity and specificity in differentiating benign versus malignant peripancreatic lymph nodes were 91.8% and 82.5%, respectively [27]. Several studies have demonstrated that EBUS elastography can differentiate between benign and malign lymph nodes.[13, 28, 29]. In a study by Izumo et al., that retrospectively included the evaluation of 75 lymph nodes, EBUS elastography showed a sensitivity, specificity, PPV, NPC and overall diagnostic accuracy of 100%, 92.3%, 94.6%, 100% and 96.7% respectively for diagnosing malignancy [12]. A NPV of 100% demonstrated that EBUS elastography could decrease the number of unnecessary aspirations and the total examination time. Controversially, other studies have indicated approximately 75% to 87% sensitivity and 65% to 68% specificity of EBUS elastography in diagnosing malignant lymph nodes [30, 31]. This discrepancy could be partly explained by different interpretations of the EBUS elastography images. For example, Izumo et al. did not include lymph nodes that appeared partially blue or nonblue on elastography images in the statistical analysis. More recently, some modifications have been applied to the observation method of elastography, i.e., a more objective quantification method. Subjective quantification should be replaced for the objective quantification method, which includes the stiffness ratio, strain histogram, stiffness area ratio, or strain ratio to predict lesion elasticity characteristics [32–35]. Fujiwara et al. [33]. achieved a sensitivity of 83.0% and a specificity of 96.2% by combining B-mode ultrasound and elastography in the prediction of nodal metastasis. However, quantitative measurement requires additional software for image processing and calculations, which are time consuming. Furthermore, additional training and experience are required to evaluate B-mode ultrasound results. Therefore, Izumo's score is currently the most widely used method in clinical practice and was applied in this study. Consistent with previous studies that found EBUS elastography sensitivity ranging from 71% to 90.6%, this study showed a sensitivity of 90.5%. However, the specificity was only 57.3%, which was less than that in other studies (67% to 82.6%) [13, 30, 36]. This discrepancy may result from multiple factors related to the final results during EBUS elastography, e.g., calcification or necrosis in the lymph nodes [3]. Previous studies have reported false positive results, mostly due to increased stiffness in cases of tuberculosis, pneumoconiosis, or sarcoidosis [37–40]. In this study, 12 lymph nodes had blue images on elastography, but the final pathological diagnosis was calcification or fibrosis. Thus, false positives did occur in this study. Conversely, necrosis, hemorrhage, or liquefaction in malignant lesions can cause false negatives. Therefore, the accuracy of the EBUS elastography score could have been affected by the structure of the lymph nodes. In addition, EBUS elastography is a subjective method and is largely related to the operator and the physiological situation (heart rate and respiratory rate) of the patient [41].

ROSE can be used to evaluate the sample during EBUS-TBNA. However, some studies have found no significant difference in the diagnostic value of EBUS-TBNA with or without ROSE [9]. ROSE can provide rapid feedback regarding sample adequacy, increasing aspiration efficiency. ROSE can also guide the operator in identifying the sampling site, determining the adequacy of the sample, and decreasing procedure duration [8]. Meena et al. showed that pulmonologists with cytopathology training could perform onsite cytological evaluation of EBUS-TBNA samples, and no significant difference in accuracy of the sample was identified when compared with cytopathologists [42, 43], benefiting clinical practice. In this study, ROSE was performed by a well-trained pulmonologist; the results demonstrate that the sensitivity and specificity of ROSE in diagnosing malignant lymph nodes were 95.73% and 79.05% respectively, consistent with findings in previous studies of 88.5% and 83.0% [43]

In previous studies, ROSE was only applied as an ancillary technique to evaluate sufficiency of the sampling in EBUS-TBNA [10, 11]. To better analyze the effects of elastography or ROSE, elastography and ROSE were combined for statistical analysis. In the present study, the sensitivity, specificity, positive likelihood ratio, and negative likelihood ratio of elastography and ROSE in diagnosing malignant lymph nodes were 85.84%, 92.65%, 11.67, and 0.15, respectively, and the AUC was 0.940; each figure was greater in the combination than in the only elastography or only ROSE group. Since EBUS elastography or ROSE during EBUS-TBNA have been applied in multiple institutions, it is possible that performing EBUS-elastography and ROSE together during EBUS-TBNA could improve its diagnostic ability.

This study had some limitations. First, it was conducted in a single institution with limited sample sizes. Further studies must be performed in larger populations. Second, this study only compared the value of the elastography score and ROSE, without additional information regarding lymph node size, integrity, or vascularity. Combining these factors could increase the diagnostic value of elastography and ROSE.

## Conclusion

The combination of elastography and ROSE during EBUS-TBNA for patients with enlarged mediastinal lymph nodes, could increase the clinical diagnostic value compared with ROSE or elastography alone. Combining elastography and ROSE during EBUS-TBNA in clinical practice is recommended.

## Supplementary Information

**Additional file 1**. The number of FN, FP, TP, TN results in three groups.

## Data Availability

The datasets used and/or analyzed during the current study are available from Jing Huang (email: hj000hj@126.com) on reasonable request.

## Abbreviations

EBUS: Endobronchial ultrasound
ROSE: Rapid onsite cytological evaluation
EBUS-TBNA: Endobronchial ultrasound-guided transbronchial needle aspiration
PPV: Positive predictive value
NPV: Negative predictive value
ROC: Receiver operating characteristic
