# Supplementary material for: Diagnostic value of endobronchial ultrasound elastography combined with rapid onsite cytological evaluation in endobronchial ultrasound-guided transbronchial needle aspiration
Source: BMC Pulm Med. 2021 Dec 20;21:423. doi: 10.1186/s12890-021-01748-4 (PMC8690901; doi:10.1186/s12890-021-01748-4)
Supplement: Supplementary file 1 — Additional file 1. The number of FN, FP, TP, TN results in three groups. [file 12890_2021_1748_MOESM1_ESM.docx]

Table 1. The number of FN, FP, TP, TN results in elastography only:

|  | | TBNA Pathology | |
| --- | --- | --- | --- |
|  |  | Malignant | Benign |
| Elastography type | 3 | **67** | **12** |
|  | 2 | **59** | **38** |
|  | 1 | **13** | **68** |

Table 2. The number of FN, FP, TP, TN results in ROSE only:

|  | | TBNA Pathology | |
| --- | --- | --- | --- |
|  |  | Malignant | Benign |
| ROSE | positive | **157** | **22** |
|  | negative | **7** | **83** |

Table 3. The number of FN, FP, TP, TN results in combination:

|  | | TBNA Pathology | |
| --- | --- | --- | --- |
|  |  | Malignant | Benign |
| Elastography type +ROSE | 3+positive | **54** | **2** |
|  | 2+positive | **43** | **3** |
|  | 1+positive | **11** | **9** |
|  | 3+negative | **2** | **7** |
|  | 2+negative | **2** | **18** |
|  | 1+negative | 1 | 29 |

Table 4. The number of TPR, TNR, FPR, FNR results in each procedure

|  | TPR | TNR | FPR | FNR |
| --- | --- | --- | --- | --- |
| Elastography type 2 considered as positive | 90.65% | 57.63% | 42.37% | 9.35% |
| Elastography type 2 considered as negative | 48.20% | 89.83% | 10.17% | 51.80% |
| ROSE | 95.73% | 79.05% | 20.95% | 4.27% |
| Combination (Elastography type 2 considered as positive) | 99.12% | 42.65% | 57.35% | 0.88% |
| Combination (Elastography type 2 considered as negative) | 97.35% | 69.12% | 30.88% | 2.65% |

TPR: true positive rate; TNR: true negative rate; FPR: false positive rate; FNR: false negative rate
